# Implementation of Total Laparoscopic Hysterectomy As the Default Technique and Lessons Learnt

**DOI:** 10.7759/cureus.16428

**Published:** 2021-07-16

**Authors:** Jose D Roman

**Affiliations:** 1 Gynaecology Department, Braemar Hospital, Hamilton, NZL

**Keywords:** total laparoscopic hysterectomy, perioperative complications, implementation of laparoscopic hysterectomy, hysterectomy in new zealand, benign gynaecolgical diseases

## Abstract

Introduction

Concerns about surgical complications and the paucity of surgical audits have been named as reasons for the slow implementation of total laparoscopic hysterectomy (TLH) in New Zealand and Australia, despite a majority of gynaecologists who would like to offer this less-invasive approach to their patients.

Material and methods

This study aims to assess the implementation of TLH as the default method of hysterectomy at a private institution in the Waikato region of New Zealand, and to identify factors related to the perioperative complications and to the failure to accomplish the above procedure laparoscopically in an unselected population.

We present 1,287 cases collected over fourteen years with an emphasis on demographics, outcomes, indications for surgery, laparoscopic completion of the surgical procedure and perioperative major complications.

Results

One hundred and fifty patients (11.7%) were nulliparous and 378 patients (29.4%) had a history of the previous laparotomy. The mean theatre time and SD was 144.84 ± 20.48 min; the mean blood loss was 137.24 ± 69 mL; the mean hospital stay was 2.07 ± 0.31 days; the median uterine weight was 177 g and the biggest uterus removed laparoscopically weighed 1,510 g. Twelve cases were converted to laparotomy (0.93%). The uterine weight had a statistically significant association with the conversion rate.

The main indications for surgery were menorrhagia and/or recurrent dysmenorrhoea in 662 patients (51.4%) and fibroid uterus in 228 patients (17.7%). Six patients (0.47%) required blood transfusions. There was a total of 74 perioperative complications (5.7%) and 16 major complications (1.24%). BMI and uterine weight had a statistically significant association with major complication rates. Seven patients (0.54%) were re-operated as a result of a complication.

Conclusion

The implementation of TLH as default is achievable and is a safe surgical option. BMI and uterine weight are factors associated significantly with major complications or conversion to laparotomy.

## Introduction

Concerns about surgical complications and the paucity of surgical audits have been named as reasons for the slow implementation [1] of total laparoscopic hysterectomy (TLH) in New Zealand and Australia, despite a majority of gynaecologists who would like to offer this less-invasive approach to their patients [2]. Despite the known advantages of laparoscopic hysterectomy when compared to abdominal hysterectomy [3] and the recommendation of the American College of Obstetricians and Gynaecologists Committee Opinion regarding minimally invasive techniques for hysterectomy [4], the implementation of this novel surgical procedure, especially TLH, when the hysterectomy is performed entirely laparoscopically, including suturing of the vaginal cuff, has been slow in Australia and New Zealand, with reports that almost 40% of hysterectomies in Australia are performed using an open surgical approach or an abdominal hysterectomy [5]. Concerns regarding surgical complications, the paucity of surgical audits [1] and lack of adequate training have motivated the development of new protocols to improve the training of young gynaecologists [6].

## Materials and methods

After having performed 412 laparoscopically assisted vaginal hysterectomies in selected patients [7], excluding patients with previous multiple laparotomies, endometrial cancer or a uterine size of more than 18 weeks gestation, from 1997 to March 2004, we introduced the technique of TLH as default for hysterectomy, but in an unselected population, from April 2004 onwards.

This study was approved by the Braemar Hospital Clinical Committee. We reviewed the outcome of 1287 patients who underwent a TLH between April 2004 and December 2018 at Braemar Hospital; which is a private regional hospital covering a population of 400,000 in the Waikato Region of New Zealand. During the same period of time, we had 88 patients with a suspected diagnosis of ovarian or advanced uterine malignancy who were offered an open procedure and therefore excluded from the study. On the other hand, we had only 13 patients who underwent a vaginal hysterectomy because of anaesthetist advice, medical contraindication for laparoscopy; uterine procidentia or the patient’s choice after preoperative counselling. Patients with early-stage endometrial cancer were included in the study.

All perioperative data were collected at the eight weeks postoperative patient consultations and the whole set of notes, including drafts and photos, were scanned and stored as portable document format files. These data were later retrieved (only one illegible case was lost because of clerical error) and analysed using the Excel for Macintosh Program (Microsoft Corp, Redmond, WA). Normally distributed data are presented as mean and standard deviation and confidence interval. Skewed data are presented as median and/or range. Categorical variables were compared using the Chi-Square/Fisher’s exact test 2 tailed, with a positive significance at p < 0.05. The odds ratio is presented together with p-values.

The following data were reported and analysed: Patient’s age, BMI (kg/m^2^), parity, indication for surgery, history of the previous laparotomy, the previous number of caesarean sections, theatre time, estimated blood loss, uterine weight (g), concomitant surgical procedures, hospital stay, intraoperative and early postoperative complications (up to eight weeks postoperative time). The primary outcomes studied were the successful completion of the laparoscopic procedure and the associated major complications.

In the cases with a history of previous surgery, only laparotomies and not laparoscopies are considered. Theatre time is defined from the time when the patient was admitted to theatre until the time when the patient left the theatre. The blood loss was estimated by examining the irrigation canister at the end of the procedure and by consensus between the surgeon, first surgical nurse assistant and the anaesthetist.

Our surgical team has always involved five members: the laparoscopic gynaecological surgeon, the first surgical nurse assistant, the anaesthetist, the Scrub nurse and the Circulating nurse. We have no second gynaecologist or gynaecologist in training to assist, but we allow visiting doctors to observe in theatre, after obtaining the patients’ approval.

We have previously reported in detail our surgical technique for laparoscopically assisted vaginal hysterectomy in 418 selected patients, operated on from 1997 to March 2004 at Braemar Hospital [7] where the main steps used were the bilateral ureteral dissection, desiccation of the uterine arteries with bipolar coagulation and the routine use of a 30-degree laparoscope and the Koh Colpotomizer system (Cooper Surgical, Trumbull, CT). Since April 2004, we have performed only TLH as default for all benign cases. We have made some technique modifications; we always mark the course of the ureters using bipolar diathermy and we dissect the ureters only if needed. We perform the skeletonization of the uterine vessels and their bipolar coagulation after the bladder peritoneum is sharply dissected off the uterus. We then use a monopolar diathermy hook to perform a circumferential colpotomy. We introduced the use of the LigasureTM laparoscopic device (Medtronic) six years ago. Uncontained uterine morcellation is performed vaginally or laparoscopically using a knife morcellator or a laparoscopic electronic morcellator (StorzR, Germany) unless we are dealing with a case with a high risk for uterine malignancy.

Following the removal of the specimen, we use polydioxanone suture (PDS) to laparoscopically close the vaginal cuff and then perform its suspension to the uterosacral ligaments. A cystoscopy is routinely performed at the end of the TLH.

We have continued with routine preoperative bowel preparation using Picoprep (Pharmatel, Pty Ltd, Thornleigh, Australia) for all patients, the routine use of a 30-degree laparoscope, a prophylactic antibiotic cover of 1 g IV Cefotetan (cephamycin-broad spectrum) shortly before the induction of anaesthesia, the Bair Hugger 250/PACU Patient Warming System (Augustine Medical, Eden Prairie, MN) and Electronic Sequential Stockings (SCD Response compression system, Tyco Healthcare, Lane Cove, Australia) until the morning after surgery. We use prophylactic subcutaneous enoxaparin sodium (ClexaneR, Sanofi-Aventis) only in patients with high risk for thrombosis. We use the RUMI system (Cooper Surgical, Trumbull, CT) for uterine manipulation unless there is uterine malignancy.

Unless there is a contraindication, we encourage the start of feeding on the same day of surgery, adequate analgesia, postoperative nausea prevention and early ambulation in all patients.

## Results

A total of 1,287 patients underwent TLH, which was completed laparoscopically in 1,275 patients (99.07%). Twelve cases (0.93%) were converted to mini-laparotomy or laparotomy. Of these 12 cases, a mini-lap procedure (using the Pelosi mini-laparotomy technique) [8] was performed in six cases, where access to the uterine arteries was precluded by cervical fibroids or lower posterior uterine fibroids (uterine weight of 962-1,500 g). One case was converted to mini-lap as the electronic morcellator broke down; two cases were converted because of a frozen pelvis and complete occlusion of the cul-de-sac; one case was converted as the view was inadequate because of severe adhesions that developed after a pelvic abscess and peritonitis; one case was converted because of difficulty on controlling the bleeding from a uterine artery and one case was converted to mini-lap to repair a moderate-sized cystotomy.

One hundred and fifty-one patients (11.7%) were nulliparous and 378 patients (29.4%) had a history of the previous laparotomy. Two hundred and nineteen patients (17%) had previously undergone between one and four caesarean sections. The demographic characteristics of the patients and the primary indications for surgery are presented in Table 1.

**Table 1:** Patients' characteristics and Indications for surgery POP - Pelvic organ prolapse

	Mean	Standard deviation
Patient age (years)	49.46	9.6
Parity	2.16	1.19
Body mass index	26.12	2.59
Theatre time (min)	144.84	20.48
Estimated blood loss (mL)	137.24	69
Hospital stay (days)	2.06	0.3
Uterine weight (g)	176.92	155.76
	N	%
Nulliparity	151	11.73
Previous laparotomy	378	29.37
Previous caesarean section	219	17.01
Indications for surgery	N	%
Menorrhagia/dysmenorrhoea	675	52.45
Fibroid uterus	228	17.71
POP/stress urinary incontinence	139	10.8
Pelvic/ovarian mass	98	7.61
Pelvic pain	58	4.5
Cervical disease	45	3.5
Endometrial hyperplasia/cancer	40	3.11
Gender reassignment	2	0.16
Breast metastatic cancer	1	0.08
Menstrual migraines	1	0.08

With regard to the main outcomes, the theatre time was 144.84 ± 20.48 minutes including other concomitant surgery performed. The average blood loss was 137.24 ± 69 mL. The mean hospital stay was 2.07 ± 0.31 days (CI: 1.46-2.68). The mean uterine weight was 176.92 g (CI: 168.42-185.42) and the biggest uterus removed laparoscopically weighed 1,510 g. The main indications for surgery were menorrhagia with/without dysmenorrhoea in 662 patients (51.4%) and multi-fibroid uterus in 228 patients (17.7%).

Six patients (0.47%) required blood transfusions: The transfusion was started electively when starting surgery in two patients, because of preoperative severe anaemia; one patient required transfusion before discharge because of postoperative symptomatic anaemia; two patients required it because of intraoperative bleeding and one patient underwent blood transfusion at three weeks after surgery because of severe gastrointestinal bleeding that occurred as a side effect of taking nonsteroidal anti-inflammatory medication.

The procedures performed concomitantly with TLH are presented in Table 2. Cystoscopy is performed routinely immediately after TLH, and uterine morcellation is placed in this table as it may substantially increase the operating time and/or complications.

**Table 2:** Concomitant surgery performed with total laparoscopic hysterectomy (*) Surgeries performed by general/plastic surgeon

	N	%
Cystoscopy	1276	99.15
Uterine morcellation	265	20.59
Laparoscopic excision of endometriosis	132	10.26
Tension-free vaginal tape procedure	125	9.71
Laparoscopic adhesiolysis	99	5.67
Vaginal pelvic floor repair with no mesh	73	0.3
Laparoscopic pelvic floor repair	56	4.35
Laparoscopic pelvic lymphadenectomies ± omental biopsies	30	2.33
Laparoscopic salpingectomies	23	1.79
Laparoscopic Burch colposuspension	22	1.71
Laparoscopic excision of large benign ovarian tumours	9	0.7
Umbilical hernia repair*	7	0.54
Vaginal mesh pelvic floor repair	5	0.39
Laparoscopic appendectomy	4	0.31
Labiaplasty	4	0.31
Laparoscopic mesh sacrocolpopexy	4	0.31
Laparoscopic cholecystectomy*	3	0.23
Vaginal myomectomy	3	0.23
Excision of benign vaginal tumour	2	0.15
Abdominoplasty*	2	0.15
Colonoscopy*	1	0.07

There were a total of 74 intraoperative and early postoperative (up to eight weeks) surgical complications (5.75%). Sixteen (1.24%) were classified as major complications: four cases of bladder injury, two of which were repaired intraoperatively and laparoscopically, one of which needed a mini-lap procedure for the repair, and one case that was missed despite normal findings on cystoscopy during surgery and a normal CT scan of the urinary tract the day after, needing a laparotomy three days later for the bladder repair. All cases of bladder damage had no history of the previous caesarean section (Table 3).

**Table 3:** Overall surgical complications

	N	%
Vaginal vault seroma/haematoma/infection	14	1.09
Port site infection	9	0.70
Urinary tract infection	9	0.70
Voiding dysfunction	8	0.62
Bladder injury	5	0.39
Partial vault dehiscence	4	0.31
Anaemia ± blood transfusion	3	0.23
Chest infection	3	0.23
Electrolyte disturbance	2	0.16
Vaginal vault granuloma	2	0.16
Pelvic pain/buttock pain	2	0.16
Ureter malfunction plus stent	1	0.08
Small bowel injury	1	0.08
Large bowel injury	1	0.08
Severe diarrhoea	1	0.08
Extraperitoneal suprapubic collection	1	0.08
Arm superficial phlebitis	1	0.08
Pelvic collection	1	0.08
Pulmonary embolism	1	0.08
Unexplained fever	1	0.08
Gastro-intestinal tract bleeding	1	0.08
Neck muscle strain	1	0.08
Ileus	1	0.08
Temporary depression	1	0.08
	74	5.75

There were also two cases of intraoperative bleeding that required laparotomy for adequate control; there was one case of postoperative severe buttock pain, probably due to pudendal nerve entrapment after an aggressive laparoscopic vaginal vault suspension, that was resolved completely after laparoscopic refashioning of the vaginal vault suspension; there was one case of unexplained pelvic pain that developed four weeks after the main surgery but a diagnostic laparoscopy was reported as negative; there was one case of small bowel injury that was repaired intraoperatively; there was one case of missed transverse colon perforation in a 140 kg patient with a 962 g uterus, which was repaired through laparotomy by a colorectal surgeon the day after; there was one case of retropubic haematoma after tape insertion, which was drained through a mini-lap under laparoscopic control; there was one case of ureteral malfunction at cystoscopy that was managed conservatively with an indwelling catheter for three weeks; one case of umbilical abscess drained in theatre under local anaesthesia and IV sedation; one case of pulmonary embolism that occurred one month after surgery; one case of postoperative ileus due to hyponatraemia and one case of vault dehiscence that was repaired vaginally under general anaesthesia. The above makes a total of seven patients (0.54%) taken back to theatre as a result of a complication.

BMI and uterine weight are the factors revealed to be associated with major complication rates. Obesity or BMI > 25 kg/m^2^ and a uterine weight > 500 g are, respectively, associated with an odds ratio of 15.61 and 6.287 to experiencing a major complication. The main factor associated with conversion to laparotomy (or mini-lap) was uterine weight > 300 g (odds ratio of 14.152) (Table 4).

**Table 4:** Risk factors for major complications and conversion to laparotomy

	Odds ratio	P-value
Obesity > 25 kg/m^2^	15.61	0.0558
Uterine weight > 500 g	6.287	0.0194
Uterine weight > 300 g	14.1525	0.001

We had three cases that needed further elective surgery as the histology revealed unexpected malignancy: Two patients with endometrial carcinoma, whom we performed laparoscopic oophorectomy and laparoscopic pelvic lymphadenectomy with no need for further radiotherapy. They both were discharged after a five-year follow-up (one of the above patients was diagnosed with Lynch syndrome one year later). One patient was diagnosed with clear cell carcinoma arising from an ovarian endometrioma; we performed laparotomy for full staging and she underwent further chemotherapy with discharge after a five-year follow-up.

## Discussion

This study reveals the outcome of TLH when offered to an unselected population and regardless of the pathology and the concomitant surgery needed. We confirmed the usefulness of TLH when factors like nulliparity (11.73%), a broad range of patients’ BMI (up to 45.1), previous surgery by laparotomy (29.37%), previous caesarean section (17%), broad uterine weight range (up to 1,510 g), excision of large ovarian benign masses (7.61%) and early endometrial cancer/hyperplasia (3.11%) were no longer a contraindication for this study.

Menorrhagia, dysmenorrhoea and fibroid uteri were the main indication for TLH. Pelvic organ prolapse (POP) was the indication in 11% of patients. Choosing TLH instead of vaginal hysterectomy for treating POP may be controversial. However, TLH is useful in the following circumstances: When there is the need for bilateral oophorectomy and/or prophylactic bilateral salpingectomies, when dealing with a substantial number of patients with previous laparotomies (29.37%) and therefore the possibility of pelvic adhesions, for cases of pelvic pain and therefore the need for a thorough pelvic examination, and in the case of patients with enlarged uteri (18% of patients had a uterine weight of more than 300 g). The usefulness of the laparoscopic approach to perform a vaginal vault suspension to the uterosacral ligaments following hysterectomy has been shown to be a safer alternative to the vaginal hysterectomy approach, reducing the risk of ureteral injury [9].

When assessing the effect of previous laparotomy (29.37%) on the conversion rate (p-value: 0.75) and its effect on the major complication rate (p-value: 0.58) we found no statistical difference, confirming that a patient who has undergone previous surgery by laparotomy is an adequate candidate for TLH [10].

The total conversion rate for TLH was 0.93% and compares favourably with other studies that have reported rates of 0.6% to 5% [11-15]. We found the conversion rate was affected mainly by the uterine weight, as in cases of uteri of more than 300 g the conversion rate was 3.88% in comparison to 0.28% for uterine weights of less than 300 g (p < 0.001), with an odds ratio of 14.15. The controversial FDA recommendation [16] in 2014 regarding the use of intraperitoneal power morcellation was a factor that increased the number of mini-laparotomies in order to perform open contained morcellations in our series. Other factors, such as age, BMI and history of previous caesarean section, did not correlate positively with the conversion rate (p > 0.05).

The overall complication rate of 5.75% is comparable to other TLH series [17,18] that report a 7%-15.4% complication rate. The major complication rate was 1.24%. Factors such as age, concomitant surgery, history of the previous laparotomy did not correlate positively with increasing complication rates (p > 0.05). A BMI > 25 kg/m^2^ and a uterine weight > 500 g correlated positively with the presence of major complications (odds ratio of 15.61 and 6.287, respectively). Out of 48 cases with a uterine weight of more than 500 g, seven cases (14.58%) were converted to mini-lap because of lower uterine or cervical fibroids making it difficult to access the uterine arteries. It is, therefore, in this group of patients with a very enlarged uterus that would present a challenge if we want to reduce further the major complication and conversion rate, at least in our experience.

This study is limited by its retrospective approach, even when the results were collected prospectively immediately after surgery. Although any study is potentially subjected to research bias we have hoped that this has been reduced when studying an unselected population. The lack of economic analysis is a drawback.

## Conclusions

This large study demonstrates that TLH is associated with a low rate of conversion to laparotomy and a low rate of major complications. It is a safe procedure as the default technique for TLH. It identifies risk factors like BMI > 25 kg/m^2^ and uterine weight > 500 g increasing the chances of major complications. It identifies the risk factor of uterine weight > 300 g increasing the chances of laparotomy and therefore important to consider when incorporating it into routine surgical practice. We hope it will encourage the sharing of surgical experience in a New Zealand setting.
